# Useful energy, economic and reduction of greenhouse gas emissions assessment of solar water heater and solar air heater for heating purposes in Gaza, Palestine

**DOI:** 10.1016/j.heliyon.2023.e16803

**Published:** 2023-05-30

**Authors:** Mohamed Elnaggar

**Affiliations:** a Laboratory of Refrigeration and Air Conditioning, Department of Engineering Professions, Palestine Technical College, Deir El-Balah, Gaza Strip 6037, Palestine; b College of Engineering and Information Technology, Israa University-Gaza, Gaza Strip 1277, Palestine

**Keywords:** Solar water heater (SWH), Solar air heater (SAH), Environmental, Pollutant emission, Greenhouse gas emissions reduction, Life cycle assessment, Sustainable development

## Abstract

This study investigated the crisis of energy from which Gaza has been suffering over the past years. It ventured to highlight the growing needs for energy and the urging need to use renewable and sustainable sources of energy such as solar thermal energy. Much specifically, it gave much importance to the solar water heater (SWH) as well as the solar air heater (SAH). These two important tools rely on clean and renewable source of energy, and their use in the Gaza Strip would greatly help in bringing about an environmental conservation and sustainable economy. The result obviously shows that both SWH and SAH systems are very suitable for space heating for buildings. The maximum annual heating energy gained is 20360.7 kWh at an inclination angle of the solar collector of 30° for SWH. While for SAH the best value of heating delivered was 19268.9 kWh at a tilt angle of 45°. Besides, the result exposes that the use of SWH and SAH systems can potentially save up to $3461.3 and $3275.7 respectively of energy cost annually. The payback achieved on the investment in SWH and SAH is 4.4 and 4 years respectively. Additionally, the utilization of SWH and SAH systems can ultimately save energy as well as potentially reduce emission of air pollution. For instance, using SWH and SAH can reduce 17306.6 and 16378.57 kg/year of CO_2_ emissions respectively.

## 1 Introduction

**Fig. 1:**
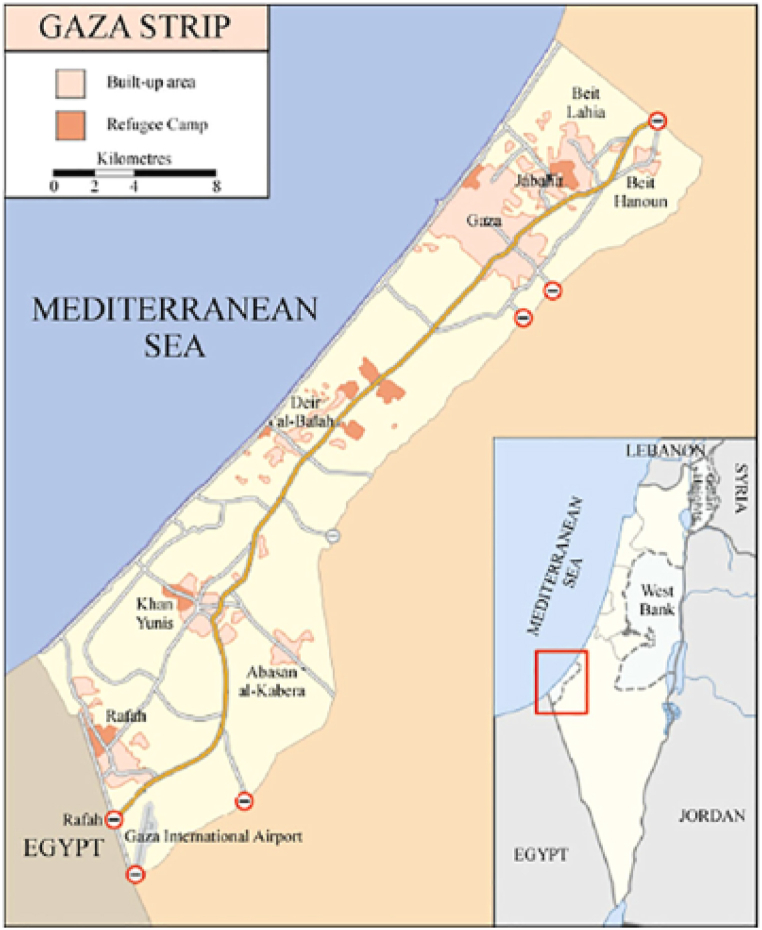
Site of Gaza Strip-Palestine [1].

Gaza Strip is an important part of Palestine. Its location on the Mediterranean Sea's eastern coast makes it an important site as shown in Fig. 1. It is a large strip that includes all the governorates locating in the southern part of Palestine, including the West Bank. Moreover, it has a distance of 6 to 12 km wide and 41 km long. Its total area is 365 square kilometers. The inhabitants of Gaza are more than 2 million according to recent statistics, and this makes it one of the most inhabited regions worldwide.

Gaza Strip's population density and the increasing number of its inhabitants is associated with an increasing demand for energy, including electricity, and a critical shortage of power supplies. The Palestinian statistics of 2009 obviously revealed this problem. In this respect, Gaza Electricity Distribution Co. (GEDCo) revealed that Gaza Strip needs electricity power of 244 MW. The report made it obvious that GEDCo supplies Gaza with 60 MW, while other power supplies contribute to 198 MW [2].

Besides, the grid's blackout schedule is averagely 12 h daily. This point evidently reflects that the current grid infrastructure in Gaza fails to provide the inhabitants with the required renewable electricity. Thus, the need for a typical supply system of energy emerges, thus suggesting the development of energy supply systems that emphasizes self-sufficiency.

In this context, solar energy can be introduced as the most sustainable source for it is clean and renewable. It is the most attractive energy source to be adopted in Gaza which is a sun-enriched region. That is, the average irradiance in Gaza is estimated about 6 KWh/m^2^ daily, on tilted 31°.

Therefore, solar thermal system might be a typical and practical solution for energy problems in Gaza. This is encouraged by the statistics reported by the PHES in July 2013. The report revealed that 62% of the Palestinian houses, which represents nearly two thirds of households in Palestine, about rely on solar heaters for heating up water. This percentage significantly contributes to saving around 600 GWh, if not more, every year, thus helping the Palestinian economy by saving more than $ 100 million per year. This point is confirmed by the Energy Authority which reveals that Palestine is among the countries that rely on solar energy. Therefore, the claim that Palestine will completely adopt solar energy system is encouraged by the already mentioned statistics.

The application of solar energy takes various forms, and this makes it a typical source of energy. These forms include photovoltaic solar panels, as the most common form, that function by converting solar radiation into electricity. As a result, the generated electricity out of this process can effectively be used to provide air-conditioning and drive electrical chillers.

Solar energy can be transformed into mechanical, chemical electrical, or thermal energy for use in a variety of applications [3] such as: solar water heating [[4], [5], [6]], heating and cooling systems [7,8], solar air heaters and space heating [9].

There are two different types of solar water heaters: (i) Flat plate collector SWHs, which consist of an insulated outer metallic box with a glass sheet on top to collect solar energy. Metallic absorber sheets that have been blackened inside have riser tubes or channels inserted into them to convey water. Solar radiation is absorbed by the absorber, which then delivers the heat to running water. (ii) Evacuated tube collector SWH – this design uses double-layer boronsilicate glass tubes that have been evacuated to serve as insulation [4].

Another significant form of solar energy is represented in solar thermal conversion. The (STE) can be directly used to get solar air conditioning which is characterized by a strong potential [10]. Thus, the use of STE for air conditioning can ultimately reduce the level of heavy reliance on the electrical grid.

Significantly, the research effort on Solar Energy has shown optimistic results about the possibility of global deploying of solar power in air conditioning. In this respect, Zhai et al. [11] have effectively designed a system and installed it. The system is concerned with using solar energy to provide air-conditioning for Shanghai Research Institute of Building Science. Furthermore, the performance of the system proved its efficacy as the average output of the refrigeration of the created air-conditioning system recorded 15.3 kW during an operating period of 8 h. Moreover, the maximum value was more than 20 kW.

Another good example in this regard can be noticed in Ref. [12]. This system is represented in the use of models with dynamic simulation. The models were applied for operating a chiller which relies on single effect absorption. The chiller was operated by using solar thermal collectors through a hot storage tank. Moreover, the models of chiller operated by being coupled either to cooling tower models or to a model that has three dimensional numerical ground heat exchanger. Such models were then validated as the researcher used data of operation of a 15 kW solar cooling system. The system was installed in the building of an office.

It is urging here to note that the traditional technology used for air-conditioning relies on electric compression chillers. Such chillers are known for peak loads during hot weather [10,13]. In order to save energy and reduce the stress on the electricity sources, absorption chillers which are thermally driven and powered by waste heat or solar energy can be used [14]. In addition, chillers can be powered by photovoltaic panels which effectively provide peak electricity for air-conditioned houses especially during hot hours.

Many scholars did comprehensive studies on solar cooling systems. In this respect, Fong et al. [15] made a comparison between five types of solar thermal cooling as well as solar electrical cooling which were used in TRNSYS; an office in Hong Kong. The researcher concluded that the two types of solar cooling reflected significant potentials of energy saving that ranged between 8.0% and 43.7%. In another study, these two solar cooling systems were examined in combination with chillers of single effect absorption. The study was conducted in two Italian locations [16].

The modern world has been using different energy forms which have ultimately played a significant role in the industrial revolution and the growth of global economy. Based on the fact that the world's reserves of fossil fuel is rapidly dwindling, many countries have resorted to sustainable energy sources, like the solar system, as the only possible option that can potentially solve the energy crisis and reduce pollution. The emphasis is made on sunlight as an abundant, sustainable and never-ending energy source that can save nature from pollution [17]. In this respect, solar collectors function as the most potential and effective method to provide thermal energy for heating purposes. This is simply done by converting solar energy into thermal one [17]. In this respect, solar air heater, shortened as (SAH), is widely used as a typical air heating system that relies on solar energy [18,19]. It is obvious that the plan to achieve the ultimate utilization of solar energy is conditioned by the complete adoption of solar air heating system.

Here, it is true to say that the absorber plate, known as the solar collector, is the SAH's most essential part. The SAH works as the absorber plate absorbs the sun radiation and converts it into the form of heat. Then the resulted heat is transferred to the air. It is noteworthy that the collector is characterized by having a low absorbing capacity of heat which evidently indicates that the circulating air is characterized by a low thermal conductivity. In this respect, traditional SAH performs very poorly. This problem can effectively be solved by increasing the heat transfer coefficient. Many scholars have introduced effective techniques to deal with the issue of SAH's poorer performance. Such techniques include extended surfaces [20], artificial roughness [21], tubular type [22,23], and different flow type [24] etc.

Another effective method to deal with this problem is represented in the development of FPSAH, which is a solar air heater with flat plate. It is covered with (CNTs) and cupric oxide nanoparticle (CuO) engrained in black paint [25]. The energy/exergy efficiencies are ultimately evaluated by using the laws of thermodynamics. Therefore, it was urging to produce a unique solar selective coating. For this purpose, researchers evaluated four airflow speeds which recorded a significant increase in the energy efficiency by approximately 24.4%. Moreover, the temperature, both inlet and outlet, across the SAH was significantly increased up to 22%.

Recently, Saxena et al. [26] presented a comparative analysis of experimental research on solar air heaters based on latent heat storage for space heating. The modified models SAHS-2 (with coconut oil) and SAHS-3 (with paraffin) are compared to a conventional model (SAHS-1), which were performed on a quality latent heat material. The results indicated that the SAHS-3 model performs best in terms of improved efficiency, increased rate of heat transfer, and net heat gain during the mild winter season.

This study is distinguished as it ventures to combine solar water heater (SWH) and solar air heater (SAH). It is also an effort to estimate the energy harvested through the two solar heating systems.

In this respect, previous studies have reflected a lack of evidence regarding the economic and environmental benefits of using SWH and SAH in Gaza. Thus, this study is mainly motivated by this point as it ventures to examine the economic as well as the environmental benefits of using SWH and SAH typical alternatives of the existing heating systems in Gaza.

## 2 Materials and methods

### 2.1 Site description

**Fig. 2:**
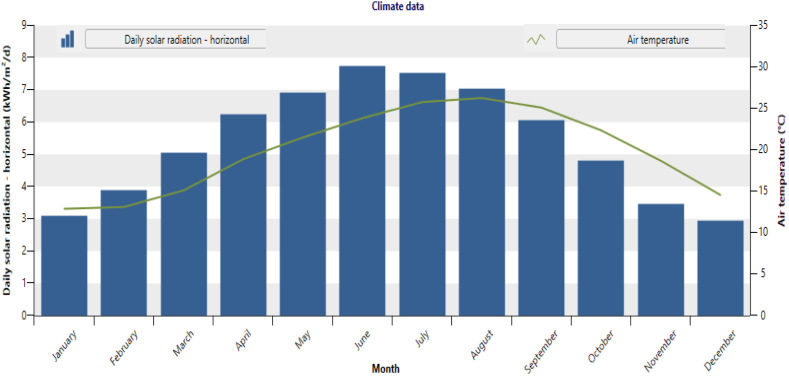
Daily solar radiation (kWh/m^2^/day) and air temperature of Gaza Strip. Data sources [29].

The site selected for this study is Gaza strip in Palestine. It is a wide area laying on the eastern coast of the Mediterranean. It is located on latitude of 31.5° N and longitude of 34.47° E [27,28]. As represented in Fig. 2 below, it is evident that the daily solar radiation-horizontal in Gaza (kWh/m^2^/d) exceeds 7 kWh/m^2^/d during the months of June, July, and August.

### 2.2 Annual energy demand and consumption for water and space heating

The annual energy demand and consumption for space heating and water heating in the Gaza Strip can differ across household and commercial sectors. The energy demand and consumption for various purposes are compared as follows:

#### 2.2.1 Household energy demand and consumption

##### 2.2.1.1 Water heating

The energy demand for water heating depends on the size of the household, how often hot water is used, and the effectiveness of the water heating system. The annual energy demand for water heating in a household can range from 2,000 to 4,000 kWh.

The energy consumption for water heating depends on the type of water heating system employed will determine the actual energy usage. Traditional electric water heaters frequently have poorer efficiencies, which leads to increased energy usage. Every year, household in the Gaza Strip spend an average of 1,500 to 2,500 kWh to heat water [30].

##### 2.2.1.2 Space heating

The energy demand for space heating in households varies significantly depending on the size, insulation, and climatic conditions of the local area. The typical annual energy need for household heating might vary from 5,000 to 10,000 kWh.

Due to the moderate temperature, space heating is a comparatively less common home need in Gaza than water heating. It is possible to employ passive solar heating methods, portable electric or gas heaters, or more conventional means like burning wood or charcoal. However, there is a lack of precise information on residential energy consumption for space heating.

#### 2.2.2 Commercial sector energy demand and consumption

##### 2.2.2.1 Water heating

The commercial sector, which includes hotels, restaurants, and public facilities, has a larger energy demand for water heating than households. Depending on the size and type of commercial establishment, it can range from 5,000 to 10,000 kWh per year, or even more, while the annual energy consumption of the commercial sector for water heating can range from 3,500 to 8,500 kWh per year, depending on the size and type of commercial establishment.

##### 2.2.2.2 Space heating

The energy demand for space heating in the commercial sector, depending on the size and kind of commercial building, might vary dramatically. When compared to smaller establishments, larger commercial facilities, such as hospitals, office complexes, or shopping centers, have higher energy demands for space heating. Annual energy demand can range from 10,000 to 30,000 kWh or higher, while annual energy consumption might vary from 8,000 to 28,000 kWh.

### 2.3 A solar water heater (SWH)

The solar thermal collector, or as it is known as the (heat exchanger), is the essence of the SWH system. This type of heat changer works by converting solar energy into another type of energy inside the transport layer [31]. The researcher, here, uses a collector with flat-plate which has a gross area of (*A*_*a*_) that equals to 24 m^2^.

**Fig. 3:**
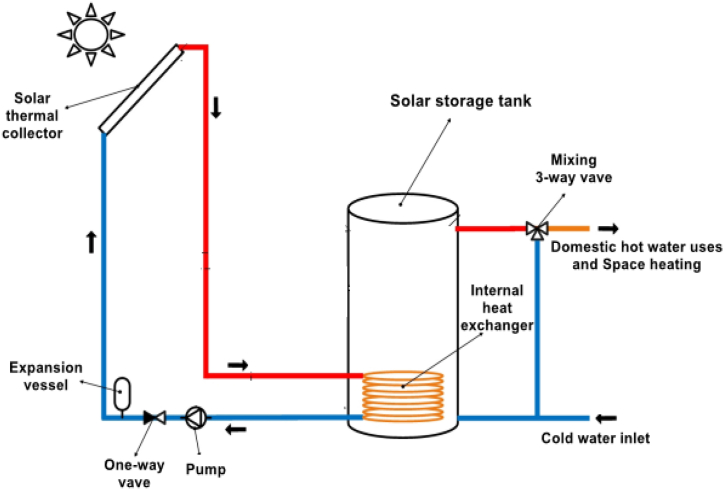
Solar water heating system [32].

As represented in Fig. 3, the (SWH) system includes a solar thermal system which is made of a number of solar collectors, pipes, a storage tank, pumps, a control unit and a cooling machine which is thermally driven. In this model of SWH, the existing solar energy, that takes the form of solar radiation flux, is ultimately utilized by a solar panel. The purpose is to produce a fluid with high temperature, which is (generally water). The hot fluid is ultimately accumulated in a fluid-storing tank devoted for this purpose.

### 2.4 Solar air heater (SAH)

The technologies of solar air heating are typical, potential, sustainable and environment-friendly as they operate by using only free, clean, and renewable energy. They not only help in defraying the increasing cost of conventional energy but contribute to saving nature from pollution. They work by simply absorbing thermal energy from direct sunlight and heating the air. The process results in heated air which can then be used to provide heat for buildings.

#### 2.4.1 Design of SAH

**Fig. 4:**
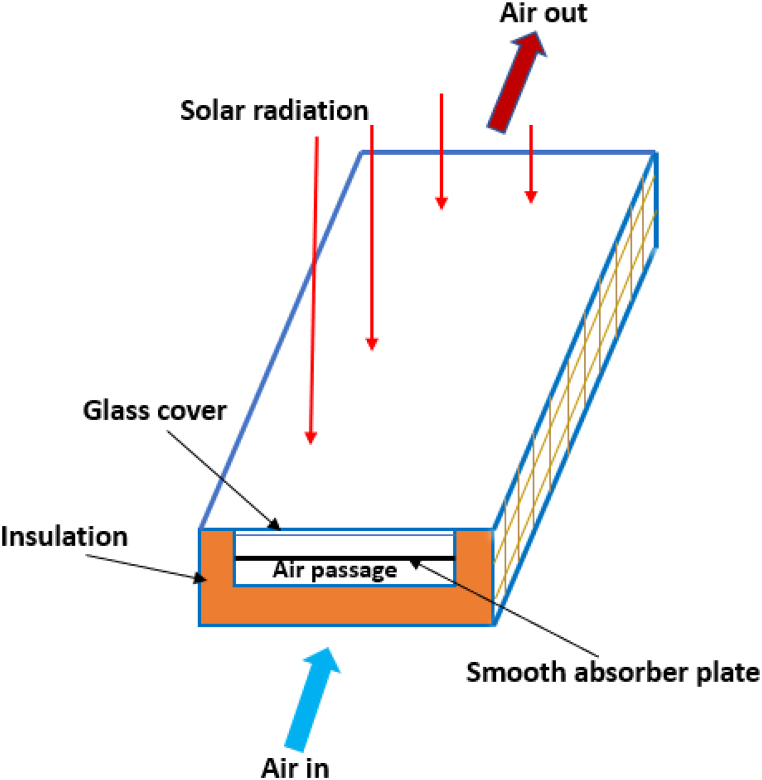
Traditional solar air heater [17].

The model of SAH is mainly designed with the use of some components that include frame, absorber – which is known as collector plate, insulation, glass cover, inlet& outlet section and black interior surfaces. The process starts with solar radiation falling on the collector plate of the (SAH) as represented in Fig. 4. The collected radiation is then absorbed and converted into heat. The resulting heat is then circulated in the air through the duct [17]. Below is a detailed description of SAH's parts [17]:

1) Frame – this part is generally made of woods or metal.
2) Absorber plate or collector – It is made of Aluminium or GI and painted black. This is intended to increase absorption of radiation to the highest level. This part gathers solar energy then circulated it in the air.
3) Matte Black Interior – in order that the optimum solar absorption is achieved, the test sections' interior surfaces are coloured black by using black matte paint.
4) Glass cover – In order to reduce the level of losing radiation from the surfaces of the absorber to the environment a glass cover is used over the absorber plate's top. The common thickness of glass covers is 3 to 4 mm.
5) Glazing – It is provided on the top surface of glass cover so as to ensure the ultimate and maximum absorption of solar radiations. This part is represented in a sheet which is made of carbonate, tempered, acrylic as well as glass materials.
6) Insulation – the purpose behind using insulation is to avoid the losses of heat from the bottom and the ducts' side. Its thickness is generally 5–8 cm. Either glass or mineral wool is used.
7) Inlet/outlet section-the purpose behind designing this part is to facilitate the Intake and discharge of air.

**Fig. 5:**
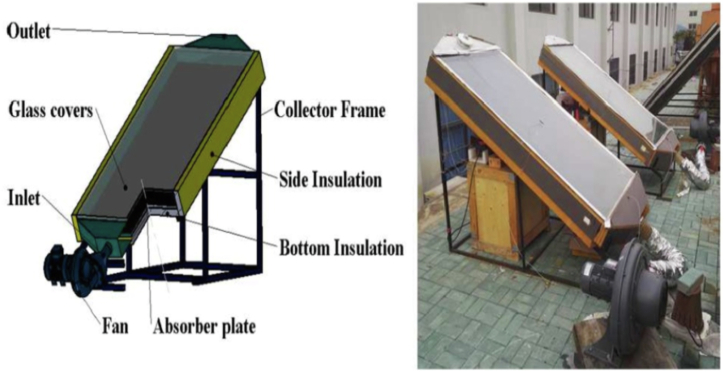
Solar air heater flat plate collector [25].

A schematic view of the solar air heater with flat plate as it is represented in Fig. 5 [25].

The common applications of solar energy in human life have included (SAHs) as significant heat exchangers [33]. Moreover, an important solar thermal application is represented in air heating. It has various functions like ventilation, space heating, as well as process heating like desalination, laundry, crop drying,…etc. [34,35]. The adoption of conventional energy for performing this process will ultimately increase cost. It will also pollute the environment. Contrastingly, the use of solar energy for various purposes, including air heating, will undoubtedly reduce cost [36].

### 2.5 Thermal performance and energy analysis of SWH and SAH

#### 2.5.1 Thermal performance of solar water heater (SWH)

The first law of thermodynamics is used to evaluate the thermal performance of solar water heaters. The thermal efficiency of solar air heater $\eta_{thw}$ is the measure of how much heat being gained by the collector from the expected total heat directed to the collector by solar incident radiation. For calculating the efficiency of the solar thermal collector ($\eta_{thw}$), the investigation relies on Equation (1) of Hottel-Whillier [19], which is also used by Refs. [[37], [38], [39]].

(1) $$\eta_{thw}=\frac{{\dot{Q}}_{uw}}{{\dot{Q}}_{c}}$$

Based on the thermodynamic analysis for the solar water system, we may say that the rate of useful heat gained, $Q_{uw}$ by the solar water system is equal to rate of heat absorbed by the water passing through the collector due to inlet and outlet temperature of water passing through the collector, $Q_{uw}$ can be determined mathematically by Equation (2) [3]:

(2) $${\dot{Q}}_{uw}={\dot{m}}_{w}C_{pw}\left(T_{wo}-T_{wi}\right)$$

However, the solar collector is exposed to a total rate incident heat (i.e., ${\dot{Q}}_{c}$) due to incident solar radiation is the multiplication of A and I_T_, then mathematically ${\dot{Q}}_{c}$ can be calculated by following Equation (3) [40,41]:

(3) $${\dot{Q}}_{c}=AI_{T}$$

Here, the ${\dot{Q}}_{uw}$ represents the gain of useable energy. Besides, the I_T_ stands as the incident of global radiation that occurs on the titled solar collector. The letter A indicates the gross area of the SWC. Furthermore, the letter ṁ_w_ indicates the rate of the water flow under operating conditions, while the C_pw_ (kJ/kg-K) represents the collector fluid's specific heat, which is water. The Two and T_wi_ (°C) denote the water temperatures, both inlet and outlet, into the collector.

Now, the thermal efficiency of solar air heater $\eta_{thw}$ is the measure of how much heat being gained by the collector from the expected total heat directed to the collector by solar incident radiation, then mathematically the thermal efficiency of the SWH ($\eta_{thw})$ can be found through the following Equation (4) [19,21,[42], [43], [44]]:

(4) $$\eta_{thw}=\frac{{\dot{m}}_{w}C_{pw}\left(T_{wo}-T_{wi}\right)}{A_{p}I_{T}}$$

#### 2.5.2 Thermal performance of solar air heater (SAH)

The SAH's thermal efficiency may be calculated using following Equation (5) [19,43]:

(5) $$\eta_{tha}=\frac{{\dot{Q}}_{ua}}{{\dot{Q}}_{c}}$$

Where ${\dot{Q}}_{ua}$ is useful heat gain from solar air collector and ${\dot{Q}}_{c}$ incident solar radiation [26,40,41]:

${\dot{Q}}_{ua}$ can be calculated by following Equation (6):

(6) $${\dot{Q}}_{ua}={\dot{m}}_{a}C_{pa}\left(T_{ao}-T_{ai}\right)$$

while ${\dot{Q}}_{c}$ can be computed by Equation (7)

(7) $${\dot{Q}}_{c}=A_{p}I_{T}$$

The SAH's thermal efficiency may be calculated by following Equation (8) [19,21,[42], [43], [44]].

(8) $$\eta_{tha}=\frac{{\dot{m}}_{a}C_{pa}\left(T_{ao}-T_{ai}\right)}{A_{p}I_{T}}$$

As m_a_ represents rate of mass flow of the air, C_pa_ stands as the air's specific heat, T_ao_ and T_ai_ (°C) represent temperatures of the air, both the inlet and outlet. Besides, I_T_ indicates the solar intensity, while A_p_ is absorption surface area.

The thermal performance of the collector can also be calculated in terms of the incident radiation [19]. Here, ${\dot{Q}}_{ua}$ and τα are given by Equations (9), (10):

(9) $${\dot{Q}}_{ua}=A_{p}\left[I_{T}F_{R}\left(\tau \alpha \right)-{F_{R}U}_{L}\left(T_{ao}-T_{ai}\right)\right]$$

(10) $$\tau \alpha =\frac{I_{bT}{\left(\tau \alpha \right)}_{b}+Id\frac{\left(1+\cos\;\beta \right)}{2}{\left(\tau \alpha \right)}_{s}+\rho I\frac{\left(1-\cos\;\beta \right)}{2}{\left(\tau \alpha \right)}_{g}}{I_{T}}$$

where *β* is tilt angle.

Now, the thermal performance of the collector can be calculated by following Equation (11):

(11) $$\eta_{tha}=\frac{{\dot{Q}}_{ua}}{A_{p}I_{T}}=F_{R}\left(\tau \alpha \right)-\frac{F_{R}U_{L}\left(T_{ao}-T_{ai}\right)}{I_{T}}$$

$F_{R}\left(\tau \alpha \right)$ is a factor used to characterize the optical efficiency of the collector, $F_{R}U_{L}$ is a factor used to characterize the collector's thermal losses [(W/m^2^)/°C] and $I_{T}$ is the global incident solar radiation on the collector [W/m^2^].

In general for glazed collectors, F_R_ (τα) is 0.68 and F_R_U_L_ is 4.90 (W/m^2^)/°C [45].

To calculate the total solar energy used by the collector $\left(I_{coll,i}\right)$ for each month (i), the following Equation (12) is used [46]:

(12) $$I_{coll,i}=I_{tilt,i}A_{coll}f_{op,i}$$

Where, *I*_*tilt,I*_ is the solar energy incident on the collector, *A*_*coll*_ is the area of collector, and *f*_*op,i*_ is operating schedule.

### 2.6 Benefit analysis of SWH and SAH

**Fig. 6:**
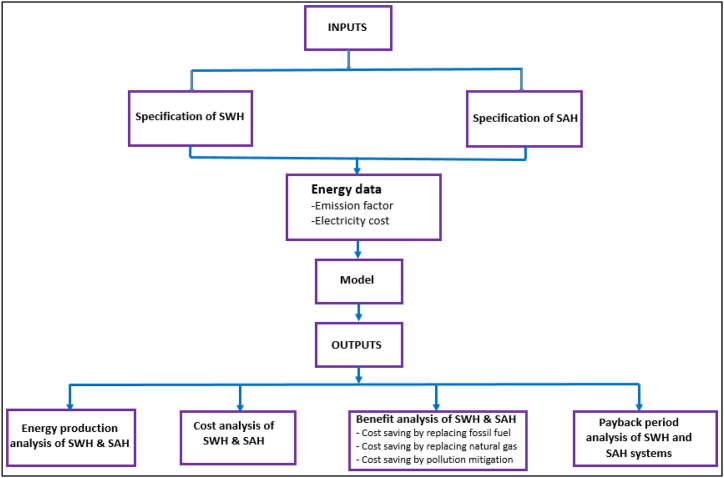
Modelling of the cost-benefit, energy production, along with payback period of SWH and SAH models.

This part includes an analysis of SWH and SAH benefit, as it is shown in Fig. 6 below. The figure embodies a simple model which is designed mainly for evaluating the cost of (SWH) and (SAH). This model embodies the key inputs for counting the potential and apparent advantages of using SWH and SAH systems in the studied location. It is also concerned with the use of this data to evaluate the cost, the energy production, benefits, as well as the return of investing the two heating systems.

Table 1 embodies the specifications and design parameters for SWH and SAH.

**Table 1:** Specifications of SWH and SAH systems.

Parameter	Unit	SWH	SAH
Solar collectors gross area	m^2^	24	24
Collector type	–	Glazed liquid flat-plate	Glazed flat-plate
Tilt angle	degree	15°, 30°, 45°, 60°, 75°	15°, 30°, 45°, 60°, 75°
Design flow rate	L/s	0.0231 L/s	1000 L/s
Design temperature	°C	60	30
Capacity	kW	16.8	16.8
F_R_ (τα) coefficient	–	0.68	0.75
F_R_U_L_ coefficient	(W/m^2^)/°C	4.9	4.9
Electricity cost	$/kWh	0.17	

#### 2.6.1 Pollutant emissions based on diesel generator energy sources

It is true that diesel fuel is a common source of pollution. Unfortunately, many electricity planets around the globe, including Gaza Strip, use diesel fuel. This is evident in Table 2 which obviously highlights the diesel fuel source's emission indicators. The table reveals that the Gaza power plant uses diesel as a sole source of fuel. It is also shown that the air impurities' emissions which are the outcome of the burning of diesel (#2 fuel oil) are basically attributed to the type of energy source. In this respect, it was obviously stated that the spread of pollutant elements that include carbon, sulfur, and nitrogen, etc. vary as per the energy source type [47].

**Table 2:** Factors of air emission according to various energy sources [[47], [48], [49], [50]].

Source of Energy	Emission factor (*EF*)kg/kWh		
	CO_2_	SO_2_	NO_x_
Diesel (#2 fuel oil)	0.850	0.0164	0.0025
Natural Gas	0.530	0.0005	0.0009

Moreover, the advantage was ultimately examined by referring to the (SWH) and (SAH) in Palestine as well as energy harvesting by the suggested SWH and SAH systems that ultimately bring about cost reduction as well as energy saving through replacing fossil fuels with renewal, potential, sustainable low-cost solar energy and emissions reduction.

The current study investigates CO_2_ emissions reduction as a result of using SWH and SAH systems instead of heating the water using an electric heater or space heating using the air conditioning device that draw electricity from conventional power plants that operate by fuel-oil and natural gas.

#### 2.6.2 Cost savings achieved by replacing non-renewable sources of energy

The saving of expenditure spent on energy can be achieved by abandoning conventional sources of energy and replacing them with other sources which are sustainable and cost-saving. This can be projected on the basis of the quantity of energy provided as well as the related costs. The energy replaced here is electricity. In this respect, the unit price is 0.17 $/kWh. To compute the expenditure which was saved by replacing energy the following equation (13) [48] is used:

(13) CBE=BE×P

Here, *BE* = total energy cut, kWh*; CBE* = cost savings from switching energy source, $*; P* = unit price of switched energy, $/kWh.

#### 2.6.3 The savings achieved through pollution reduction

The reduction of pollution can bring about cost saving, and the saved cost that is achieved by decreasing gas emissions can ultimately be appraised by the decrease in the level of air pollutants by using solar energy. In this respect, Jing et al. (2015) [48] stated that the estimated expenses for treating 1000 kg of CO_2_, NO_x_ and SO_x_ is approx. $ 20, $ 674.5, and $ 656.5, respectively. The assessment of cost conserving by gas emission reductions was done by using Equations (14), (15)):

(14) $$BP_{p,f}=BE\times EF_{p,f}\times PE_{f}$$

(15) $$CBP_{f}=\sum_{p}(BP_{p,f}\times TE_{p})$$

Here, EF_p,f_ = pollutant's emission factor p by source of energy f, kg/kWh; PE_f_ = energy source percentage f in the energy mix; BP_p,f_ = quantity of emissions p that are decreasing (e.g. CO_2_, NO_x_ and SO_x_) if SWH and SAH avoid utilizing electricity generated by energy source f, kg; CBP_f_ = avoided cost related to treating greenhouse gas.

### 2.7 An analysis of the total cost of SWH and SAH systems

Since the economical profit associated with SWH and SAH systems was ultimately valued every year, it was natural that the essential capital and costs of operation were obviously anticipated annually for the payback analysis afterward. This point is elaborated in Table 3 which embodies the cost of SWHs and SAHs in Gaza Strip.

**Table 3:** Cost of SWH and SAH panel.

Energy Systems	Life period (Year)	Size of the solar collectors (m^2^)	Initial cost $/m^2^	Total initial cost $	Maintenance cost $/yr	Total Maintenance cost $	Total Cost $
SWH	15	24	600	14400	120	1800	16200
SAH	15	24	520	12480	120	1800	14280

### 2.8 Analysis of simple payback period

The duration of reimbursement refers to the period which is needed after starting using SWH and SAH to regain its financial investment. Moreover, this period was ultimately by making a comparison between benefit and annual cost over certain duration. The annual cost, in this study, related to each working year was ultimately examined, paying attention to the operational cost and the capital. Furthermore, the economic profit was ultimately examined on the basis of annual energy cost saved.

## 3 Results and discussion

### 3.1 SWH and SAH gained energy

According to the first law of thermodynamics, energy can only be changed from one form to another and cannot be created or destroyed. In the case of solar water heater (SWH) and solar air heater (SAH), they employ heat energy, which is created when sunlight is converted into energy, to heat water or air. According to the second law of thermodynamics, energy is wasted as unusable waste heat as a result of heat flowing from hot to cold. SWH and SAH are designed to enhance heat transfer to the water or air being heated while minimizing heat loss to the environment. Here, it can be said that the thermodynamic interpretation of the system's efficiency is a measurement of how well a system transforms solar energy into heat energy that can be used to heat water or air.

**Fig. 7:**
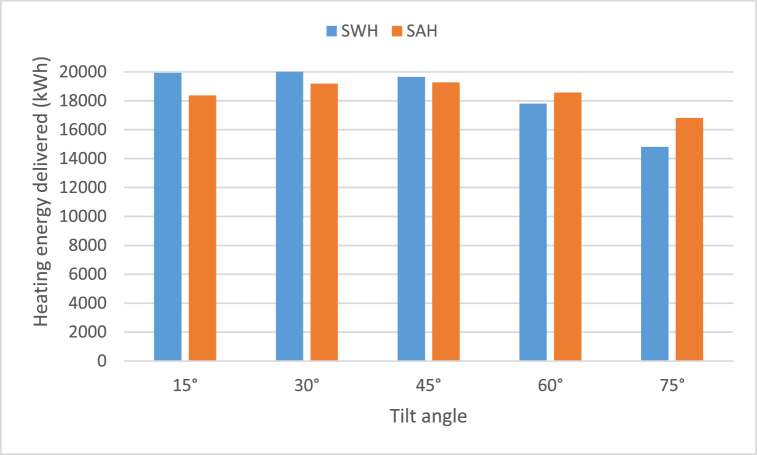
Annual heating energy delivered for SWH and SAH.

Specifications and design parameters values are previously shown in Table 1. The researcher has used glazed flat-plate collector for SWH and SAH which has gross area of 24 m^2^. It has been applied at the tilt angles of 15°, 30°, 45°, 60°, and 75°. As represented in Table 4, Table 5, Table 6, Table 7, Table 8, the lowest heating delivered was recorded in the months of December and January. On the other hand, the maximum useful energy was recorded in the months of July and August. The maximum annual useful energy received was 20360.7 kWh at an inclination angle of the solar collector of 30° for SWH as shown in Table 5 as well as in Fig. 7. While for SAH the best value of heating delivered was 19268.9 kWh at the tilt angle of 45° as shown in Table 6 as well as in Fig. 7.

**Table 4:** Monthly heating energy delivered from SWH and SAH at tilt angle of 15°.

Months	Daily solar radiation Horizontal (kWh/m^2^/d)	Daily solar radiation Tilted (kWh/m^2^/d)	Heating delivered (kWh)		Solar thermal efficiency η_th_	
			SWH	SAH	SWH	SAH
January	3.09	3.83	1038.9	1786.7	0.365	0.627
February	3.91	4.52	1179.7	1901.7	0.388	0.626
March	5.05	5.52	1667.4	2572.6	0.406	0.626
April	6.25	6.45	1911.3	2909.9	0.412	0.627
May	6.91	6.81	2058.8	2688.2	0.406	0.531
June	7.76	7.46	2150.9	0	0.400	0
July	7.52	7.31	2172.2	0	0.399	0
August	7.03	7.13	2103.8	0	0.397	0
September	6.07	6.52	1863.8	856.4	0.397	0.182
October	4.81	5.56	1633	1991.2	0.395	0.481
November	3.46	4.25	1152.4	1918.2	0.377	0.627
December	2.94	3.74	988.8	1743.3	0.355	0.627
Annual	**5.4**	**5.76**	**19921**	**18368.2**	**0.391**	**0.413**

**Table 5:** Monthly heating energy delivered from SWH and SAH at tilt angle of 30°.

Months	Daily solar radiation Horizontal (kWh/m^2^/d)	Daily solar radiation Tilted (kWh/m^2^/d)	Heating delivered (kWh)		Solar thermal efficiency η_th_	
			SWH	SAH	SWH	SAH
January	3.09	4.37	1237.1	2038	0.381	0.627
February	3.91	4.89	1298.1	2059.5	0.395	0.627
March	5.05	5.7	1727.2	2659.7	0.407	0.627
April	6.25	6.32	1874.9	2852.2	0.412	0.627
May	6.91	6.4	1945	2540.1	0.408	0.534
June	7.76	6.87	2001.1	0	0.405	0
July	7.52	6.79	2037.6	0	0.403	0
August	7.03	6.86	2035.5	0	0.399	0
September	6.07	6.63	1891.3	850.7	0.396	0.178
October	4.81	6.01	1766.4	1991.2	0.395	0.445
November	3.46	4.81	1339.2	2172.2	0.387	0.627
December	2.94	4.34	1207.3	2022.2	0.374	0.626
Annual	**5.4**	**5.8325**	**20360.7**	**19185.8**	**0.397**	**0.410**

**Table 6:** Monthly heating energy delivered from SWH and SAH at tilt angle of 45°.

Months	Daily solar radiation Horizontal (kWh/m^2^/d)	Daily solar radiation Tilted (kWh/m^2^/d)	Heating delivered (kWh)		Solar thermal efficiency η_th_	
			SWH	SAH	SWH	SAH
January	3.09	4.67	1342.8	2176.7	0.386	0.626
February	3.91	5.01	1334.6	2108.9	0.396	0.626
March	5.05	5.6	1693.0	2609.4	0.406	0.626
April	6.25	5.87	1744.1	2650.2	0.413	0.627
May	6.91	5.68	1730.6	2394.2	0.410	0.567
June	7.76	5.93	1743.9	0	0.408	0
July	7.52	5.93	1796.7	0	0.407	0
August	7.03	6.24	1868.3	0	0.403	0
September	6.07	6.38	1826.9	845.1	0.398	0.184
October	4.81	6.13	1801.7	1991.3	0.395	0.437
November	3.46	5.11	1433.1	2305.2	0.390	0.627
December	2.94	4.69	1331.6	2187.9	0.382	0.627
Annual	**5.4**	**5.60**	**19647.3**	**19268.9**	**0.399**	**0.412**

**Table 7:** Monthly heating energy delivered from SWH and SAH at tilt angle of 60°.

Months	Daily solar radiation Horizontal (kWh/m^2^/d)	Daily solar radiation Tilted (kWh/m^2^/d)	Heating delivered (kWh)		Solar thermal efficiency η_th_	
			SWH	SAH	SWH	SAH
January	3.09	4.71	1356.1	2195.1	0.387	0.626
February	3.91	4.86	1289.2	2047.5	0.395	0.627
March	5.05	5.2	1564.1	2426	0.404	0.627
April	6.25	5.14	1515.6	2318.5	0.410	0.627
May	6.91	4.73	1422.2	2206.6	0.404	0.627
June	7.76	4.79	1394.8	0	0.404	0
July	7.52	4.84	1459.8	0	0.405	0
August	7.03	5.31	1593.7	0	0.403	0
September	6.07	5.8	1667.4	838.8	0.399	0.200
October	4.81	5.92	1740.4	1991.2	0.395	0.452
November	3.46	5.12	1435.8	2309.8	0.390	0.627
December	2.94	4.78	1362.4	2230.7	0.383	0.627
Annual	**5.4**	**5.1**	**17801.5**	**18564.2**	**0.398**	**0.420**

**Table 8:** Monthly heating energy delivered from SWH and SAH at tilt angle of 75°.

Months	Daily solar radiation Horizontal (kWh/m^2^/d)	Daily solar radiation Tilted (kWh/m^2^/d)	Heating delivered (kWh)		Solar thermal efficiency η_th_	
			SWH	SAH	SWH	SAH
January	3.09	4.48	1277.1	2090.3	0.383	0.627
February	3.91	4.46	1161.9	1878.4	0.388	0.627
March	5.05	4.55	1339.2	2121.1	0.396	0.627
April	6.25	4.18	1191.7	1885.1	0.396	0.626
May	6.91	3.62	1021.1	1687.4	0.379	0.627
June	7.76	3.48	943.1	0	0.376	0
July	7.52	3.59	1016.7	0	0.381	0
August	7.03	4.19	1224.7	0	0.392	0
September	6.07	4.92	1406.2	830.3	0.397	0.234
October	4.81	5.38	1579.8	1991.2	0.395	0.497
November	3.46	4.84	1347.4	2183.9	0.387	0.627
December	2.94	4.6	1300.2	2146.1	0.380	0.627
Annual	**5.4**	**4.36**	**14809.1**	**16813.8**	**0.3875**	**0.4266**

**Fig. 8:**
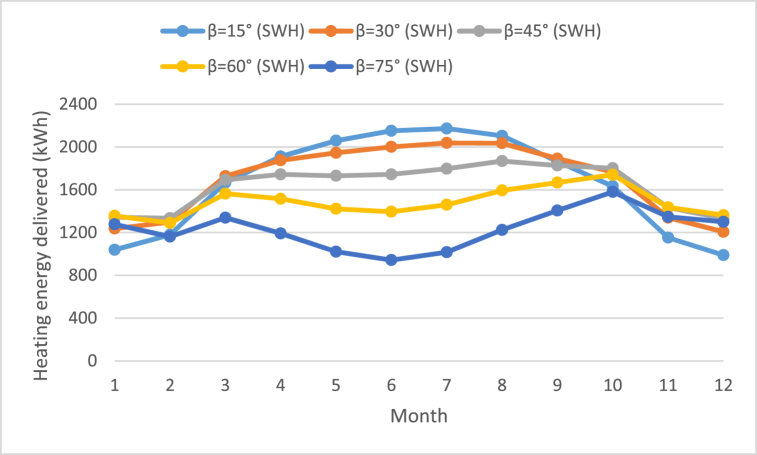
Average monthly heating energy delivered for SWH at various tilt angles.

**Fig. 9:**
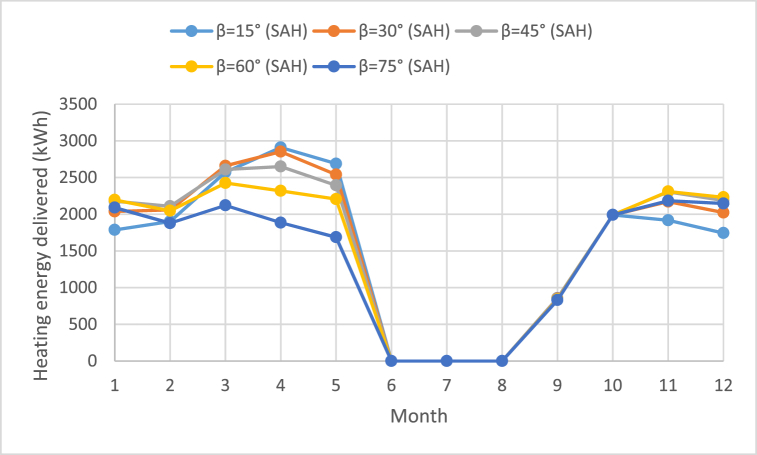
Average monthly heating energy delivered for SAH at various tilt angles.

As represented in following Tables, the highest level of monthly heating energy was gained in July; 2172.2 kWh at tilt angle of 15° for SWH as shown in Fig. 8. Moreover, for SAH, Table 4, Table 5, Table 6, Table 7, Table 8 as well as in Fig. 9 reflect that June, July and August, recorded zero value of energy. This is because these months record high temperatures which require no heating but cooling.

It should be noted here that due to variations in solar radiation and outside temperature, solar water heater and solar air heater efficiencies vary throughout the year. The efficiency of solar water heaters is typically better in the summer since there is more sunshine, the air is warmer, and there is less heat loss. In contrast, solar air heaters perform more effectively in the winter when the outside temperature is lower, resulting in less heat loss and a greater temperature difference between the heated air and the outside air.

Furthermore, it is reflected in Fig. 8 that the value related to gained energy recorded an increase in summer months which showed the maximum useful energy, reaching; 2150.9 and 2172.2 kWh at tilt angle of 15° in the months of June and July respectively. However, the gained energy decreased during winter as it is evident in the maximum energy value recorded during December and January; 1362.4 and 1356.1 kWh respectively at tilt angle of 60°.

Fig. 9 shows the average monthly heating energy delivered for SAH at various tilt angles. It is clear from Fig. 9 that the best useful energy was obtained in the spring season is at angles of 15° and 30°, while in the fall and winter season it is at inclination angle of 60°. In addition, it is noticeable in the summer months June, July and August, recorded zero value of heating delivered. This is because these months record high temperatures which require no heating but cooling.

It is obvious that with the exception of the summer months, the monthly heating energy produced by the system of SAH is truly greater than that produced by the SWH. However, in addition to the common feature of using both SWH and SAH systems in space heating, the SWH lies in the fact that it is also possible to benefit from heating water in the whole year for domestic use.

### 3.2 Benefit analysis of SWH and SAH systems

The use of (SWH) and (SAH) systems have great benefits which are evaluated on the basis of total quantity of energy produced by SAH and SWH including profit. The most common benefits are represented in swapping energy sources as well as pollution reduction. In this respect, solar collectors area was taken 24 m^2^ for each of the SWH and SAH systems, so as to provide heating for a small building. Table 3 lists the costs of SWH and SAH specification.

#### 3.2.1 Cost savings through substituting energy sources

It is estimated that nearly all of the building appliances in Gaza rely on electricity which is generated from non-sustainable, non-renewable and imported fossil fuels [50]. In this respect, the utilization of solar energy, great benefits regarding emission and reduction in energy cost can effectively be attained. Scholars have highlighted this point by stating that cost saving can be obtained by altering energy source, as it is evident in Pan et al. [51]. It is conditioned by the quantity of energy generated as well as its expenses once solar energy is adopted.

**Fig. 10:**
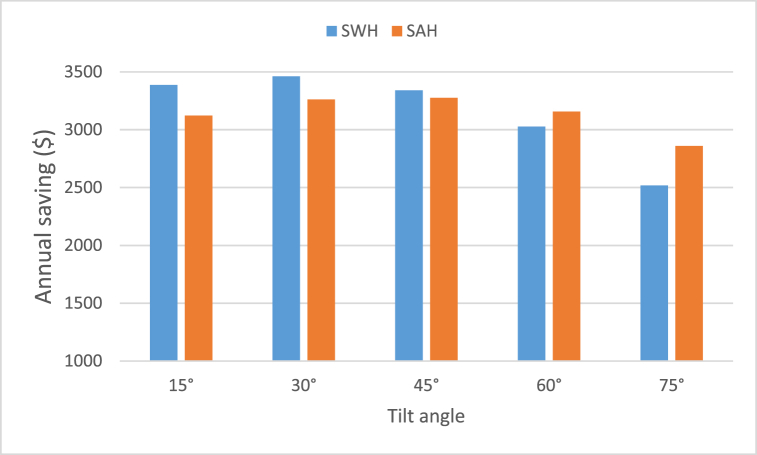
Annual savings as a result of using SWH and SAH at various collector tilt angles.

To his argument is proved in the present study, as indicated in Table 4, Table 5, Table 6, Table 7, Table 8 regarding SWH and SAH, which highlights the energy saved by shifting to the solar system. The maximum annual useful energy received was 20360.7 kWh which can effectively be produced at tilt angle of 30° for SWH as shown in Table 5. As a result, a conventional SWH can ultimately avoid spending an approximate amount of $3461.3 of energy fee annually, as represented in Fig. 10. (Electricity cost is $0.17/kWh).

**Fig. 11:**
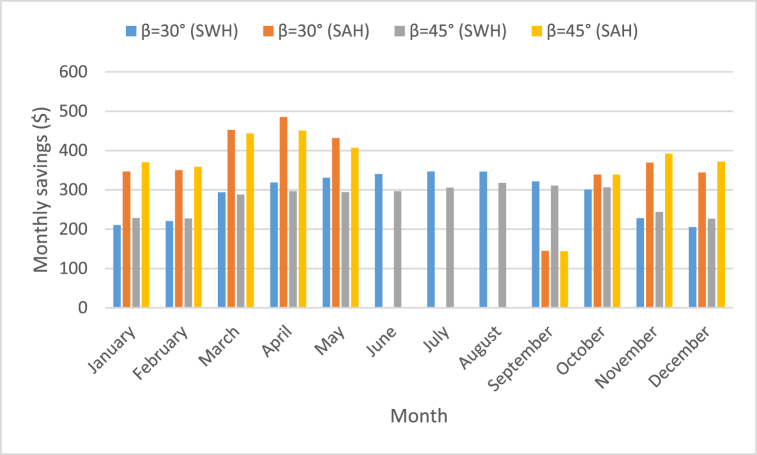
Monthly savings due to the use of SWH and SAH at tilt angles of 30° and 45°.

The values regarding the energy saved by the use of SAH, as represented in Table 6, reveals that the maximum annual energy gained recorded 19268.9 kWh/year which can effectively be produced at tilt angle of 45°. Therefore, a conventional SAH can effectively avoid spending an approximate amount of $3275.7 of energy fee annually, as represented in Fig. 10. Besides, the estimated monthly savings distribution is represented in Fig. 11 for both SWH and SAH. The values reflect a potential cost-effective benefit to the community, particularly for households with low-income levels. This is absolutely true as it can effectively and potentially decrease the financial inconvenience which is caused by electricity bills.

#### 3.2.2 Pollutant emissions based on power plants that operate by fuel-oil and natural gas

The adoption of the SWH and SAH systems can bring about great benefits through their potential in reducing air emissions. It is true that these two heating systems do not consume electrical energy. Moreover, the process of using solar water and air energies as a substitute to fossil fuels brought about an indirect reduction of the GHGs emissions [50]. It has been proven that the decrease in emissions of air pollutants leads to a noteworthy decrease in expenses, and the costs of treating impurities have been estimated using the two equations (14), (15).

In this respect, Table 2 represents the emission factors for electricity generation in Gaza Strip, Palestine. As it is exemplified in Table 2, the CO_2_ emission is ultimately the highest. This indicates that generating electricity using coal, diesel or natural gas releases large amounts of CO_2_ compared to other air pollutants.

In the current study, two cases are analyzed to estimate CO_2_ emissions reduction as a result of using SWH and SAH systems instead of heating the water using an electric heater or space heating using the air conditioning device that draw electricity from conventional power plants that operate by Ref. [52]:

Case 1 fuel-oil.

Case 2 natural gas.

**Fig. 12:**
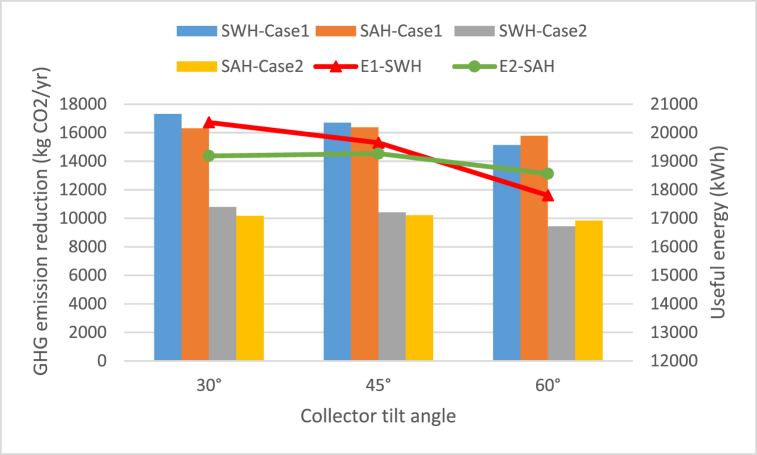
GHG emissions reduction and useful energy by using SWH and SAH for case 1 and case 2.

The reduction in GHGs emissions by using SWH and SAH instead of electricity produced by oil burning power plant (case 1) and natural gas burning power plant (case 2) are represented in Fig. 12. In general, the decrease in GHG emissions in case 1 is greater than that in case 2. This is because the use of oil (diesel) to generate electricity results in more CO_2_ emissions than the use of natural gas.

Therefore, when using SWH and SAH for heating purposes instead of using diesel-generated electricity, it works to reduce gas emissions more than using natural gas to produce electricity. For case 1, the maximum annual emission reduction (kg/yr) of CO_2_, SO_2_, and NOx are 17306.6, 333.92, and 50.9 kg/year by using SWH at collector tilt angle of 30° and 16378.57, 316.01, and 48.17 kg/year using of SAH respectively at collector tilt angle of 45°. Whereas in case 2, the maximum annual emission reduction (kg/yr) of CO_2_, SO_2_, and NOx are 10791.17, 10.18, and 18.3 kg/year by using SWH at collector tilt angle of 30° and 10212.52, 9.63, and 17.34 kg/year using of SAH respectively at collector tilt angle of 45°.

#### 3.2.3 Cost savings by emissions reduction

Paying attention to the approximate treatment, the fees for 1 ton of CO_2_, NOx, and SO_2_ are $20, $674.5, and $656.5. In this respect, the expenses prevented as a result of the reduction in GHGs emissions by using SWH and SAH instead of electricity produced by oil burning power plant (case 1) are represented in Table 9. As represented in Table 9, the maximum annual emission reduction (kg/yr) of CO_2_, SO_2_, and NOx are 17306.6, 333.92, and 50.9 kg/year by using SWH at collector tilt angle of 30° and 16378.57, 316.01, and 48.17 kg/year using of SAH respectively at collector tilt angle of 45°. The emission treatment's cost that can potentially and effectively be avoided annually recorded $604.77 by SWH and $572.35 by SAH.

**Table 9:** Reduction of pollutant emissions by using SWH and SAH instead of electricity produced by oil burning power plant (case 1).

Energy sources	Tilt angle	Useful energy (kWh)	Emission reduction (kg/yr)			Cost saving ($/yr)			Total savings ($/yr)
			CO_2_	SO_2_	NO_x_	CO_2_	SO_2_	NO_x_	
SWH	30°	20360.7	17306.60	333.92	50.90	346.13	225.23	33.42	604.77
	45°	19647.3	16700.21	322.22	49.12	334.00	217.33	32.25	583.58
	60°	17801.5	15131.28	291.94	44.50	302.63	196.92	29.22	528.76
SAH	30°	19185.8	16307.93	314.65	47.96	326.16	212.23	31.49	569.88
	45°	19268.9	16378.57	316.01	48.17	327.57	213.15	31.63	572.35
	60°	18564.2	15779.57	304.45	46.41	315.59	205.35	30.47	551.41

Table 10 presents the reduction in GHGs emissions by using SWH and SAH instead of electricity produced by natural gas burning power plant (case 2). The maximum annual emission reduction (kg/yr) of CO_2_, SO_2_, and NOx are 10791.17, 10.18, and 18.3 kg/year by using SWH at collector tilt angle of 30° and 10212.52, 9.63, and 17.34 kg/year using of SAH respectively at collector tilt angle of 45°. The emission treatment's cost that can potentially and effectively be avoided annually recorded $234.72 by SWH and $222.13 by SAH.

**Table 10:** Reduction of pollutant emissions by using SWH and SAH instead of electricity produced by natural gas burning power plant (case 2).

Energy sources	Tilt angle	Useful energy (kWh)	Emission reduction (kg/yr)			Cost saving ($/yr)			Total savings ($/yr)
			CO_2_	SO_2_	NO_x_	CO_2_	SO_2_	NO_x_	
SWH	30°	20360.7	10791.17	10.18	18.32	215.82	6.87	12.03	234.72
	45°	19647.3	10413.07	9.82	17.68	208.26	6.63	11.61	226.50
	60°	17801.5	9434.80	8.90	16.02	188.70	6.00	10.52	205.22
SAH	30°	19185.8	10168.47	9.59	17.27	203.37	6.47	11.34	221.18
	45°	19268.9	10212.52	9.63	17.34	204.25	6.50	11.39	222.13
	60°	18564.2	9839.03	9.28	16.71	196.7805	6.26	10.97	214.01

In this context, reduction of Greenhouses emissions is consistent with the Paris agreement which emphasized the necessity of constructing Greenhouses for the success of SDGs.

### 3.3 Life cycle assessment (LCA)

LCA, which is an acronym for Life Cycle Assessment (LCA), is considered a product life cycle assessment, sometimes called Life Cycle Analysis (LCA). It is an assessment of the impact of the product on the surrounding environment in conjunction with the presence of energy and during the life cycle of the product from the beginning of its production to its end. In the case of this research paper, the LCA analysis may include several elements, for example; the cost of air and water heaters, their operating costs, their lifespan, and the impacts of all the previous elements on the environment (here are their impacts on the environment by measuring CO_2_ levels, greenhouse gas levels, and greenhouse gases). Additionally, the cost of pipes for the heating cycle in the solar water and air heaters.

Some previous studies established the total cost of equipment (C_s_) used in solar water and air heaters through the following equation [4,19,53]:

$$C_{s}=C_{a}A_{c}+C_{i}$$

where Ca, Ac, and Ci are area-dependent costs, collector area, and area-independent costs, respectively.

The results for the economic costs used in solar water and air heaters are shown in Table 3. The results for environmental impacts are shown in Table 9, Table 10.

### 3.4 Cost investigation and payback period (PBP)

**Fig. 13:**
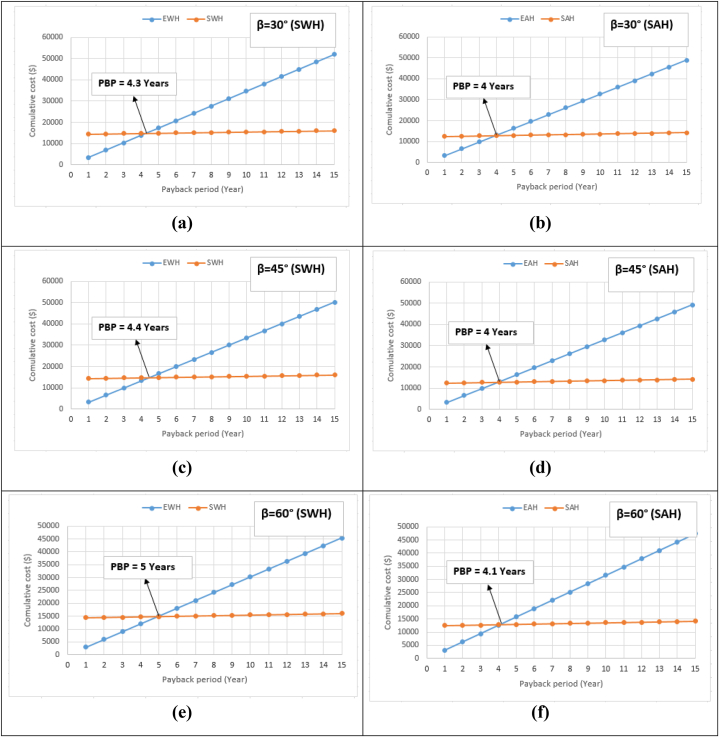
Payback period (PBP) for SWH and SAH at various collector tilt angles: (a) SWH at 30°, (b) SAH at 30°, (c) SWH at 45°, (d) SAH at 45°, (e) SWH at 60°, and (f) SAH at 60°.

The cost of investment generally includes cost of initial installation, cost of operation – that includes energy consumption -, and expense of maintenance. In this respect, systems of SWH are known for their economic nature. That is, they never incur energy costs. Thus, the total cost of investment includes the solar system installation's initial cost and the cost of maintenance [54]. In order to guarantee the achievement of SWH's high productivity, SH systems require maintenance at least once a year. Therefore, it is ultimately urging to identify the entire servicing as well as to repair cost for each operation year. In this respect, Fig. 13 provides a comparison between the SWH's cumulative costs and EWH's cumulative costs for 15 years at various collector tilt angles. SWHs which can potentially and effectively be operated for a longer period reflect a lower cost per year than those worked for a shorter working time. Since longer operating time denotes that the cost of investment can ultimately be regained back over longer time period, it is important to make sure that the SH system can effectively and potentially operate for longer time, otherwise it will incur much cost. The investment payback period, with regard to SWH in Gaza, was ultimately performed and reported in this research. Accordingly, the economic benefits of applying SH systems can ultimately recoup the initial cost of investment. This is effectively achieved through the savings obtained by reducing expenditure on electrical energy. In this regard, environmental benefit is not examined as a cost to the SWH system. This is because it represents the funds which are reimbursed by the government or industry, mainly for treating electricity production's impurities. Basically, the investment's payback can eventually be checked by simply creating a parallel between the SWH systems' costs and benefits. For calculating the investment's payback period, the annual cost's curves along with annual profit were ultimately constructed. Furthermore, the investment's payback period corresponds to the operation year in which the annual cost's curves and yearly profit intersect. As represented in Fig. 13, the annual SWH's cost decreased significantly with the operation year, as the yearly benefit goes high with the operation year. Besides, the period of payback denotes the operation year in which the year's curves; the yearly cost and yearly benefit intersect. The best payback achieved on the investment in SWH and SAH are 4.4 and 4 years respectively. Details are represented in Fig. 13.

### 3.5 Optimum solutions for sustainable development

**Fig. 14:**
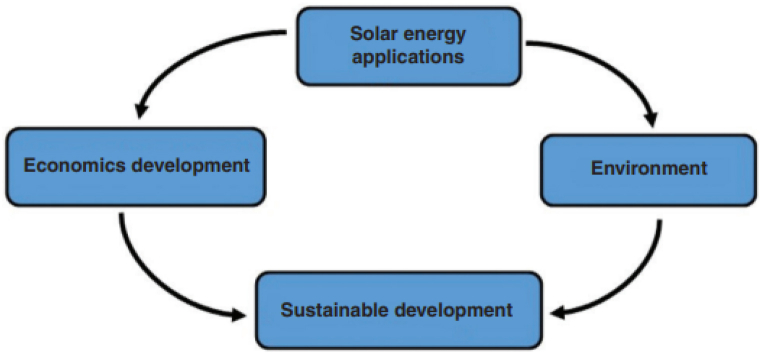
Solar energy applications cycle for achieving sustainable development [56].

Solar energy plays an important role in achieving and promoting sustainable development [55], and this is represented by two axes as shown in Fig. 14:

The first is from an economic point of view, which is obtaining clean, environmentally friendly energy that is available to all and is inexhaustible by converting sunlight into thermal or electrical energy and reducing the use of fossil fuels that are depleted and pollute the environment.

The second is through the environmental aspect of reducing CO_2_ and greenhouse gas emissions.

The optimum solution in this study is through the use of SWH and SAH, as the maximum annual heating energy gained is 20360.7 kWh at an inclination angle of the solar collector of 30° for SWH. While for SAH the best value of heating delivered was 19268.9 kWh at a tilt angle of 45°. Besides, the result exposes that the use of SWH and SAH systems can potentially save up to $3461.3 and $3275.7 respectively of energy cost annually. The minimum period of payback achieved on the investment in SWH and SAH is 4.4 and 4 years respectively. Additionally, the use of SWH and SAH can reduce 17306.6 and 16378.57 kg/year of CO_2_ emissions respectively.

### 3.6 Households' culture for energy consumption pattern of water and space heating

In the Gaza Strip, household cultures and energy consumption patterns for water and space heating are influenced by various factors, including cultural practices, availability of resources, economic conditions, and technological advancements.

#### 3.6.1 Cultural practices

Bathing Culture: Cleanliness is highly prized in Gaza, and hot water for bathing is greatly valued. This creates a high demand for water heating, particularly during the cooler months.

Extended Family Structures: Many Gaza households feature extended family structures, with numerous generations living in the same dwelling. This frequently leads to higher energy usage for space heating in order to accommodate larger living spaces.

#### 3.6.2 Current energy consumption patterns

Water Heating: Currently, most Gaza families rely on electric or solar water heaters to generate hot water. However, due to limited resources and high energy costs, some households may resort to archaic methods such as stove-top water heating.

Space heating: Households frequently rely on portable gas or electric heaters, as well as traditional techniques such as burning wood or utilizing charcoal, for space heating. These methods, however, can be ineffective, costly and contribute to indoor air pollution.

#### 3.6.3 Future projection and scenarios

1. Renewable Energy Integration: Increasing the use of renewable energy sources, such as solar power, for water heating and space heating in Gaza is one possible future scenario. This would necessitate the purchase of solar water heating devices as well as solar panels for energy generation.
2. Energy efficiency measures: Adoption of energy-efficient technology such as heat pumps, efficient insulation, and energy-saving appliances to minimize energy consumption for water heating and space heating is another scenario.
3. Improved infrastructure: Infrastructure investments, such as the expansion of electricity grids and the implementation of district heating systems, can help optimize energy use and save costs.
4. Behavioral change and education: Public awareness campaigns and educational initiatives promoting energy-saving methods can encourage households to utilize more sustainable energy.

## 4 Conclusion

The Palestinian energy sector is unreasonably and completely dependent on imports to meet the energy supply's scarcity. These imports are derivatives of fossil fuels. This represents a serious problem as fossil fuels are costly and harmful to the environment. Thus, it was urging to adopt cost-saving and environment-friendly energy sources. One effective and potential alternative to decrease the reliance on fossil fuels utilization and overcome ecological challenges is to shift to using SWH systems as substitutes of electric water heaters.

It is noticeable through the use of SWH and SAH systems that both can be used in domestic buildings, but SAH can be used for space heating in the cold months that record low outside temperatures.

It is noticeable through the use of the air and water systems that both can be used in domestic buildings, but air heating can be used in the cold months that record low outside temperatures. As for SWH, in addition to using it for space heating during the winter months, it can be used in domestic uses that need water heating throughout the year.

The study result indicates that both SWH and SAH systems are very suitable for space heating for buildings. The maximum annual heating energy gained is 20360.7 kWh at an inclination angle of the solar collector of 30° for SWH. While for SAH the best value of heating delivered was 19268.9 kWh at a tilt angle of 45°. Besides, the result exposes that the use of SWH and SAH systems can potentially save up to $3461.3 and $3275.7 respectively of energy cost annually. The payback achieved on the investment in SWH and SAH is 4.4 and 4 years respectively. Additionally, the utilization of SWH and SAH systems can ultimately save energy as well as potentially reduce emission of air pollution. For instance, using SWH and SAH can reduce 17306.6 and 16378.57 kg/yr of CO_2_ emissions respectively. The results emphasized that replacing of EHS by SWH and/or SAH are promising. Moreover, it is consistent with the United Nations SDGs as it can effectively and potentially contribute to global energy security, economic development, and sustainable environment.

## Author contribution statement

Mohamed Elnaggar: Conceived and designed the experiments; Performed the experiments; Analyzed and interpreted the data; Contributed reagents, materials, analysis tools or data; Wrote the paper.

## Data availability statement

Data will be made available on request.

## Declaration of competing interest

The authors declare that they have no known competing financial interests or personal relationships that could have appeared to influence the work reported in this paper.
